# Sli15^INCENP^ Dephosphorylation Prevents Mitotic Checkpoint Reengagement Due to Loss of Tension at Anaphase Onset

**DOI:** 10.1016/j.cub.2010.06.023

**Published:** 2010-08-10

**Authors:** Lesia Mirchenko, Frank Uhlmann

**Affiliations:** 1 Chromosome Segregation Laboratory, Cancer Research UK London Research Institute, 44 Lincoln's Inn Fields, London WC2A 3PX, UK

**Keywords:** CELLBIO

## Abstract

The mitotic checkpoint, also known as the spindle assembly checkpoint, delays anaphase onset until all chromosomes have reached bipolar tension on the mitotic spindle [1–3]. Once this is achieved, the protease separase is activated to cleave the chromosomal cohesin complex, thereby triggering anaphase. Cohesin cleavage releases tension between sister chromatids, but why the mitotic checkpoint now remains silent is poorly understood. Here, using budding yeast as a model, we show that loss of sister chromatid cohesion at anaphase onset would engage the mitotic checkpoint if this was not prevented by concomitant separase-dependent activation of the Cdc14 phosphatase. Cdc14, in turn, inactivates the mitotic checkpoint by dephosphorylating Sli15^INCENP^, a subunit of the conserved Aurora B kinase complex that forms part of the proposed chromosomal tension sensor. Dephosphorylation-dependent relocation of Sli15^INCENP^ from centromeres to the central spindle during anaphase is seen in organisms from yeast to human [4–8]. Our results suggest that Sli15^INCENP^ dephosphorylation is part of an evolutionarily conserved mechanism that prevents the mitotic checkpoint from reengaging when tension between sister chromatids is lost at anaphase onset.

## Highlights

► Loss of cohesion at anaphase onset can reengage the mitotic checkpoint ► This is prevented by activation of the Cdc14 phosphatase at the same time ► Cdc14 inactivates the mitotic checkpoint by dephosphorylating Sli15^INCENP^ ► A conserved mechanism inactivates the mitotic checkpoint in anaphase

## Result and Discussion

### Reengagement of the Mitotic Checkpoint Due to Loss of Tension in Anaphase

During chromosome alignment on the mitotic spindle, a single chromosome that has not yet come under bipolar tension is sufficient to delay mitotic progression [1–3]. Only when all chromosomes are bioriented, the mitotic checkpoint is silenced, leading to activation of the anaphase promoting complex (APC), a ubiquitin ligase complex. The APC now ubiquitinates, and thereby primes for degradation, the anaphase inhibitor securin, as well as mitotic cyclins. Securin destruction liberates the protease separase to trigger sister chromatid separation by cleaving the chromosomal cohesin complex while cyclin destruction downregulates mitotic cyclin-dependent kinase (Cdk) activity to promote mitotic exit. As sister chromatids split, the cohesive counterforce required for the build-up of tension on the metaphase plate is lost from all chromosomes. Reengagement of the mitotic checkpoint at this stage would inhibit the APC, stabilize securin and cyclins again, and thus impede further mitotic progression [9]. Why the ubiquitous loss of tension at anaphase onset goes undetected by the checkpoint remains poorly understood. One possibility is that the viscous drag of chromosomes on their way to the spindle poles substitutes for tension between sister chromatids, but this has not been experimentally addressed.

**Figure 1:**
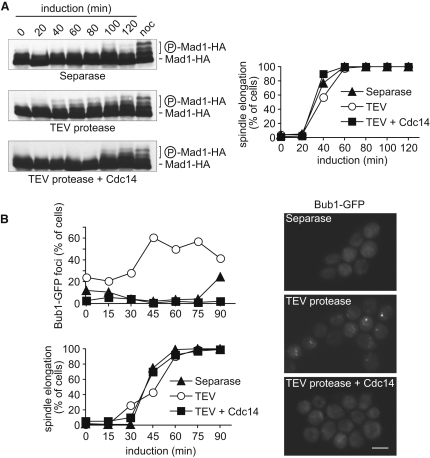
Cdc14 Prevents Mitotic Checkpoint Engagement Due to Loss of Sister Chromatid Cohesion at Anaphase Onset (A) Cells were arrested in metaphase by Cdc20 depletion, and expression of separase, TEV protease, or TEV protease together with Cdc14 was induced. Activation of the mitotic checkpoint was monitored by the phosphorylation-induced electrophoretic mobility shift of Mad1, fused to a HA-epitope tag to facilitate western detection. The same cells treated with the spindle poison nocodazole (5 μg/ml; noc), but uninduced, served as a positive control for mitotic checkpoint activation. (B) As in (A), but checkpoint activation was visualized by the appearance of Bub1-GFP nuclear foci. Images are of cells 45 min after induction; scale bar represents 5 μm. Anaphase spindles of 4 μm or longer were scored as elongated. See also Figure S1.

We set out to investigate whether loss of cohesion at anaphase onset would, in principle, reengage the mitotic checkpoint, and if so, how this is normally prevented. We studied budding yeast cells arrested in metaphase by depletion of the APC activator Cdc20. In these cells, we initiated anaphase onset by ectopic expression of either separase or the foreign TEV protease that also triggered loss of cohesion by cleaving accordingly engineered cohesin [10]. Mitotic checkpoint signaling was monitored by the phosphorylation status and kinetochore recruitment of the checkpoint components Mad1 and Bub1, respectively [11, 12]. Mad1 phosphorylation, accompanied by retarded electrophoretic mobility, a sign for checkpoint engagement, was not detectable during separase-triggered anaphase (Figure 1A), consistent with the notion that the mitotic checkpoint remains silent. Only at later time points, some Mad1 phosphorylation became apparent, which was probably the consequence of progression into the next cell cycle after separase expression [13]. In contrast, when anaphase onset was triggered by TEV protease expression, Mad1 became phosphorylated concomitant with anaphase onset. Similarly, recruitment of Bub1 into distinct nuclear foci, a marker for recognition of tensionless kinetochores by the checkpoint, was observed at the time of anaphase onset in response to TEV protease expression, but not after separase expression (Figure 1B). This suggests that loss of cohesion at anaphase onset results in a loss of tension that is, in principle, detected by the mitotic checkpoint, but an activity of separase, different from cohesin cleavage, prevents this. These observations are consistent with a recent report that the checkpoint protein BubR1 associates with anaphase chromosomes after TEV protease-induced cohesin cleavage in mitotically arrested *Drosophila* embryos [14].

### Cdc14 Prevents Reengagement of the Mitotic Checkpoint during Anaphase

In addition to splitting sister chromatids, separase promotes activation of the Cdc14 phosphatase, a key Cdk opponent during budding yeast mitotic exit [13, 15]. To address whether Cdc14 makes cells insensitive to loss of tension at anaphase onset, we ectopically coexpressed Cdc14 with TEV protease in metaphase-arrested cells. This prevented both Mad1 phosphorylation and Bub1 foci formation in response to sister chromatid splitting (Figures 1A and 1B), indicating that Cdc14 can inactivate the responsiveness of the mitotic checkpoint to loss of tension. Ectopic Cdc14 expression also overcame a mitotic arrest induced by the spindle depolymerizing drug nocodazole (see Figure S1 available online), further emphasizing its capacity to inactivate the mitotic checkpoint.

**Figure 2:**
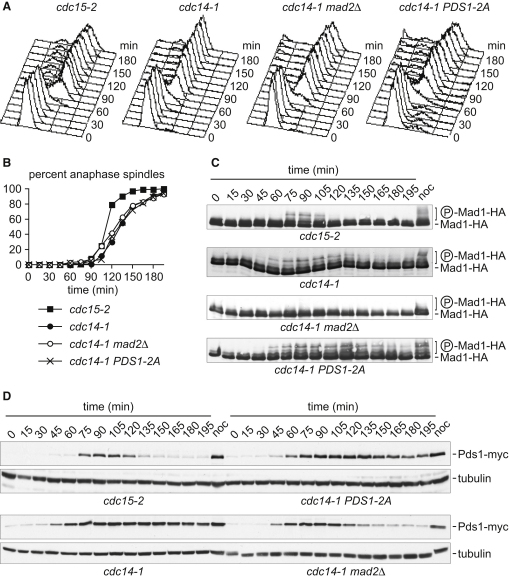
Persistent Mitotic Checkpoint Signaling in *cdc14-1* Mutant Anaphase Cells (A) Cells of the indicated genotypes were released from α-factor block in G1 into synchronous cell cycle progression at nonpermissive temperature (37°C) for the *cdc14-1* and *cdc15-2* alleles. Cell cycle progression was monitored by fluorescence-activated cell sorting (FACS) analysis of DNA content. (B) Spindles of 4 μm or longer were scored as elongated. (C) The Mad1 phosphorylation status in cells from the above experiment was analyzed by western blotting. (D) Levels of securin (Pds1), fused to a myc epitope tag to facilitate detection, were analyzed by western blotting. Tubulin served as a loading control. See also Figure S2.

To confirm that Cdc14 is responsible for restraining the checkpoint in anaphase, we examined a *cdc14-1* temperature-sensitive strain. As a control, we used *cdc15-2* mutant cells that, like *cdc14-1* cells, arrest in telophase at restrictive temperature but activate Cdc14 in early anaphase [15]. After synchronization in G1 using α-factor, both strains progressed through the early stages of the cell cycle with similar kinetics (Figure 2A). Anaphase spindle elongation started at the same time but took longer to complete in the case of *cdc14-1* cells, most likely because of the Cdc14 requirement for stable spindle midzone formation, as described previously [5, 16–18] (Figure 2B). In *cdc15-2* control cells, Mad1 phosphorylation became detectable at the time of S phase and disappeared again at the metaphase-to-anaphase transition (Figure 2C). In contrast, Mad1 phosphorylation persisted long into anaphase in *cdc14-1* cells, indicating a failure to inactivate the mitotic checkpoint. Checkpoint engagement during anaphase is expected to inhibit the APC and consequently stabilize securin. Consistently, we observed high levels of securin in *cdc14-1*, but not *cdc15-2*, anaphase cells (Figure 2D). The persistence of securin was due to the mitotic checkpoint in *cdc14-1* cells, because it was no longer observed after deletion of the gene encoding the checkpoint component Mad2. Anaphase spindle elongation was not advanced in *cdc14-1* cells lacking Mad2, confirming that the rate of spindle elongation was affected by Cdc14 independently of mitotic checkpoint regulation.

The above results suggest that the mitotic checkpoint is engaged in *cdc14-1* anaphase cells. However, checkpoint silencing and securin destruction are thought to be a prerequisite for anaphase onset. Persistent Mad1 phosphorylation and securin in *cdc14-1* cells might therefore be the consequence of checkpoint reengagement after it had initially been satisfied. A transient decrease in Mad1 phosphorylation and securin levels might have been obstructed by the limited mitotic synchrony of our cell population after release from α-factor block. When we performed a similar experiment with cells synchronized at the metaphase-to-anaphase transition by depletion and reinduction of Cdc20, transient securin destruction and checkpoint-dependent reaccumulation became obvious in *cdc14-1*, but not *cdc15-2*, cells (Figure S2). These observations suggest that Cdc14 is required to prevent mitotic checkpoint reengagement in anaphase.

It has been suggested that Cdc14 promotes securin destruction during anaphase by direct securin dephosphorylation. However, introduction of a nonphosphorylatable securin allele, *PDS1-2A*, that is no longer protected from degradation by Cdk phosphorylation [19] did not avert securin stabilization in *cdc14-1* anaphase cells (Figure 2D). This is in contrast to the marked dependence of securin stabilization on Mad2, suggesting that securin accumulation in *cdc14-1* mutant anaphase is primarily the consequence of the checkpoint.

### Cdc14 Overcomes a Mitotic Checkpoint-Dependent Cell Cycle Delay

**Figure 3:**
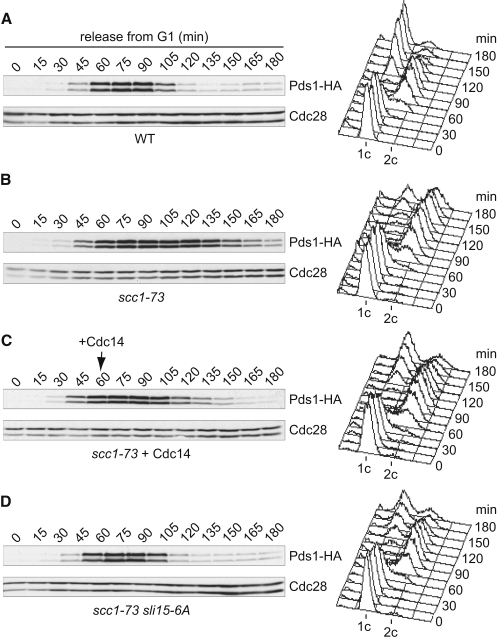
Cdc14 Relieves the Mitotic Checkpoint Delay Due to Absence of Tension (A and B) Wild-type (A) and *scc1-73* (B) cells were grown in YP medium containing raffinose as carbon source, arrested in G1 using α-factor, and released into synchronous cell cycle progression at restrictive temperature (35°C). α-factor was added back at 75 min for rearrest in the following G1. Cell cycle progression was monitored by FACS analysis of DNA content and western blotting against securin (Pds1) fused to a HA-epitope tag. Cdc28 served as a loading control. (C) In a second *scc1-73* culture, Cdc14 expression from the *GAL1* promoter was induced by galactose addition at 60 min. (D) A third *scc1-73* culture carried the *sli15-6A* allele. See also Figure S3.

The above experiments have analyzed markers of the checkpoint and have suggested that Cdc14 is required to prevent checkpoint reengagement due to loss of tension at anaphase onset. The important physiological consequence of checkpoint signaling is a mitotic delay. We were unable to analyze a checkpoint-mediated delay to mitotic progression in *cdc14-1* anaphase cells because of the essential requirement of Cdc14 for mitotic exit independently of checkpoint inactivation. To explore the potential of Cdc14 as a checkpoint regulator, we therefore analyzed its impact in a setting where mitosis is delayed in cells that fail to establish tension between sister chromatids as a result of defective sister chromatid cohesion. As described [20], securin destruction and progression through mitosis was delayed in cells carrying the temperature-sensitive cohesin subunit *scc1-73* (Figures 3A and 3B). Ectopic Cdc14 expression in *scc1-73* cells largely overcame the delay to both securin destruction and mitotic progression (Figure 3C). This demonstrates that Cdc14 can override a mitotic checkpoint delay due to absence of tension between sister chromatids. Whereas in this experiment Cdc14 overcame the checkpoint response to lack of tension in prometaphase, Cdc14 would normally disable the response to loss of tension in early anaphase, its normal time of activation.

### Sli15^INCENP^ Dephosphorylation Inactivates the Mitotic Checkpoint

How does Cdc14 inactivate the mitotic checkpoint? It has been suggested that APC-dependent degradation of the Mps1 kinase disables the checkpoint in anaphase [21]. Mps1 degradation is in part mediated by the APC activator Cdh1, whose binding to the APC requires dephosphorylation by Cdc14. However, Cdh1 activation is a late event during mitotic exit, and, consistently, we found that Mps1 levels declined only late and gradually in anaphase (Figure S3). Mps1 degradation may therefore not act fast enough to render the mitotic checkpoint insensitive to loss of tension at anaphase onset. Furthermore, Mps1 remained stable, whereas the mitotic checkpoint was efficiently inactivated in response to separase expression in mitotically arrested cells (Figure S3). These observations suggest that Cdc14 inactivates the mitotic checkpoint by a different or additional mechanism.

A candidate Cdc14 substrate for checkpoint inactivation is Sli15^INCENP^. It forms part of the conserved Aurora B kinase complex at centromeres, required for conveying lack of tension to the mitotic checkpoint [20]. Its Cdc14-dependent dephosphorylation at anaphase onset mediates Sli15^INCENP^ relocation from centromeres to the spindle midzone [5, 22]. To investigate the consequences of Sli15^INCENP^ dephosphorylation, we employed cells carrying the *sli15-6A* allele in which six Cdk phosphorylation sites have been mutated, mimicking a dephosphorylated state independently of Cdc14 [5, 16]. *sli15-6A* cells were unable to delay mitosis in response to defective sister chromatid cohesion in *scc1-73* cells (Figure 3D). This suggests that Sli15 phosphorylation is a prerequisite for its mitotic checkpoint function and that its dephosphorylation, which normally occurs in anaphase, inactivates the checkpoint.

The Sli15-6A protein was proficient in its essential function in chromosome biorientation on the mitotic spindle [23] (Figure S4), as well as in the mitotic checkpoint response to spindle depolymerization by nocodazole (Figure S1). Sli15-6A therefore appears to separate the functions of the Aurora B kinase complex in (1) the mitotic checkpoint response to loss of tension and (2) the error correction of kinetochore microtubule attachments and the checkpoint response to nocodazole treatment. This separation of function could be quantitative in nature, relating to reduced *sli15-6A* kinetochore levels as a result of its premature relocation to the mitotic spindle [5]. Error correction may require lower levels of the Aurora B kinase compared to generation of the mitotic checkpoint signal in response to loss of tension. A mitotic checkpoint function of the Aurora B kinase complex, independently of generating unattached kinetochores, has previously been documented in vertebrates and fission yeast [24–27].

**Figure 4:**
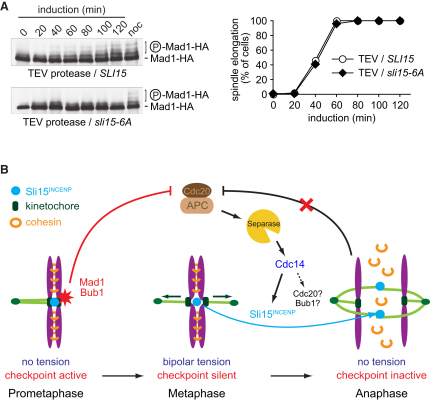
Nonphosphorylatable Sli15-6A Prevents Mitotic Checkpoint Reengagement in Anaphase (A) Budding yeast cells harboring wild-type *SLI15* or the *sli15-6A* allele were arrested in metaphase by Cdc20 depletion. Loss of sister chromatid cohesion was triggered by TEV protease expression. Mad1 phosphorylation and anaphase spindle elongation were monitored as in Figure 1. (B) Model for mitotic checkpoint inactivation in anaphase. During chromosome alignment on the mitotic spindle in prometaphase, a mitotic checkpoint signal, including the Mad1 and Bub1 proteins, emanates from kinetochores that have not yet come under tension. This prevents APC activation by Cdc20. Generation of the checkpoint signal depends on the physical proximity between the Aurora B kinase complex and its targets on tensionless kinetochores. Once bipolar tension is established in metaphase, the checkpoint is silenced and the APC degrades securin to activate separase. Cohesin cleavage now triggers anaphase, and tension is lost again from kinetochores. This would reactivate the checkpoint, but this is prevented by Sli15^INCENP^ dephosphorylation and consequent relocation of the Aurora B kinase complex to the spindle midzone. Dephosphorylation of additional Cdk targets might contribute to maintain an inactive checkpoint. See also Figure S4.

We finally tested whether Sli15^INCENP^ dephosphorylation is indeed sufficient to prevent mitotic checkpoint engagement when tension between sister chromatids is lost at anaphase onset. We induced sister chromatid separation in metaphase-arrested *sli15-6A* cells by TEV protease expression. Unlike in *SLI15* control cells, in which Mad1 became phosphorylated at the time of anaphase onset, this response was no longer observed in *sli15-6A* cells (Figure 4A). This suggests that Sli15 dephosphorylation turns off the ability of cells to respond to loss of tension between sister chromatids at anaphase onset. We note that despite compromised anaphase A movement, kinetochores do not obviously lose microtubule attachment during TEV protease-triggered anaphase [16]. This is consistent with the possibility that centromere retention of the Aurora B kinase complex reengages the mitotic checkpoint in anaphase without activating error correction, possibly because of the kinetochore microtubule geometry at this time.

### A Conserved Mechanism to Inactivate the Mitotic Checkpoint in Anaphase

We show here that loss of tension between sister chromatids at anaphase onset, caused by cleavage of cohesin, would in principle reengage the mitotic checkpoint. This is prevented in budding yeast by concomitant activation of the Cdc14 phosphatase, which, helped by cyclin proteolysis and Cdk downregulation, leads to dephosphorylation of Sli15^INCENP^. The Aurora B kinase complex is an integral part of the mitotic checkpoint also in vertebrates [24–26]. Its sudden relocation from the inner centromere to the spindle midzone, promoted by INCENP dephosphorylation, is a hallmark feature of this “chromosomal passenger” complex [4, 6–8]. In an accompanying study, Vázquez-Novelle and Petronczki ([28], this issue of *Current Biology*) show that relocation of the Aurora B kinase complex is required to prevent untimely checkpoint protein recruitment to human kinetochores in anaphase, suggesting that a conserved mechanism prevents the mitotic checkpoint from reengaging in anaphase.

The resulting model of Aurora B kinase regulation as part of the mitotic checkpoint is illustrated in Figure 4B. The spatial proximity between inner centromeric Aurora B kinase and not-yet-identified phosphorylation targets at the outer kinetochore, probably including Ndc80, is thought to initiate checkpoint signaling [29, 30]. Once biorientation is achieved, the kinetochore undergoes a conformational change in response to the exerted physical tension [31, 32]. This increases the distance between Aurora B kinase and the outer kinetochore and brings its phosphorylation targets out of reach [23, 29]. Protein phosphatase 1, resident at the outer kinetochore, now removes the phosphoepitopes and thereby silences the checkpoint [27, 33, 34]. At anaphase onset, however, kinetochores revert to their tensionless conformation [31]. This would bring the outer kinetochore back into proximity of Aurora B kinase, leading to reengagement of the mitotic checkpoint. We propose that Sli15^INCENP^ dephosphorylation and consequent dissociation of the Aurora B kinase complex from centromeres prevents unscheduled checkpoint reactivation at this time.

In addition to Sli15^INCENP^, a number of other mitotic checkpoint components undergo cell cycle regulation. Cdk-dependent phosphorylation of fission yeast Bub1 and vertebrate Cdc20 are required for a functional mitotic checkpoint [35–37]. The dephosphorylation timing of these proteins during mitotic exit and the phosphatases responsible remains to be characterized. Although Sli15^INCENP^ dephosphorylation inactivates the mitotic checkpoint at the very source of the checkpoint signal, we speculate that dephosphorylation of these additional targets, as well as Mps1 degradation [21], contributes to inactivate the mitotic checkpoint at anaphase onset and to keep it inactive until well into the next cell cycle. This ensures that loss of tension, which causes a robust block to mitotic progression in prometaphase, will not impede mitotic exit and return to G1 once the signal to the separation of sister chromatids has been given.

## Experimental Procedures

### Yeast Strains and Techniques

Details of the yeast strains used in this study can be found in Table S1. Genes were fused at their endogenous gene loci with affinity epitope tags for western blot detection or a 3xGFP cassette for detection by fluorescent microscopy using polymerase chain reaction products. Arrest of cells in metaphase by depletion of Cdc20 under control of the *MET3* promoter and expression of separase, TEV protease, or Cdc14 under inducible control of the *GAL1* promoter were as described previously [10, 16]. Analysis of Mad1 phosphorylation was performed by electrophoresis of whole cell extracts and prepared using an alkaline extraction method [38] on low crosslinking SDS-polyacrylamide gels (8%; acrylamide to bisacrylamide ratio 33.5:0.3), followed by western blotting. Antibodies used for western detection were α-HA clone 12CA5, α-myc clone 9E10, α-Clb2 serum (sc-9071), α-PSTAIRE serum recognizing Cdc28 (sc-53, both Santa Cruz Biotechnology), α-tubulin antibody clone YOL1/34 (AbD Serotec), and α-actin serum (ab8227, Abcam).

### Microscopy

Cells expressing Bub1-3xGFP were fixed in 100% ethanol and mounted on 2% agarose pads for examination. Recruitment to kinetochores was confirmed by its colocalization with Ndc80-RFP (data not shown). Spindle elongation was analyzed in formaldehyde-fixed cells by indirect immunofluorescence using α-tubulin antibody clone YOL1/34. Fluorescent images were acquired using an Axioplan 2 imaging microscope (Zeiss) equipped with a 100× (NA = 1.45) Plan-Neofluar objective and an ORCA-ER camera (Hamamatsu).

## Supplemental Information

Document S1. Four Figures and One Table
