# Supplementary material for: Sli15INCENP Dephosphorylation Prevents Mitotic Checkpoint Reengagement Due to Loss of Tension at Anaphase Onset
Source: Curr Biol. 2010 Aug 10;20(15):1396–401. doi: 10.1016/j.cub.2010.06.023 (PMC2964898; doi:10.1016/j.cub.2010.06.023)
Supplement: Document S1. Four Figures and One Table [file mmc1.pdf]

Current Biology, Volume *20*

Supplemental Information

Sli15^{INCENP} Dephosphorylation Prevents Mitotic Checkpoint Reactivation Due to Loss of Tension at Anaphase Onset

Lesia Mirchenko and Frank Uhlmann

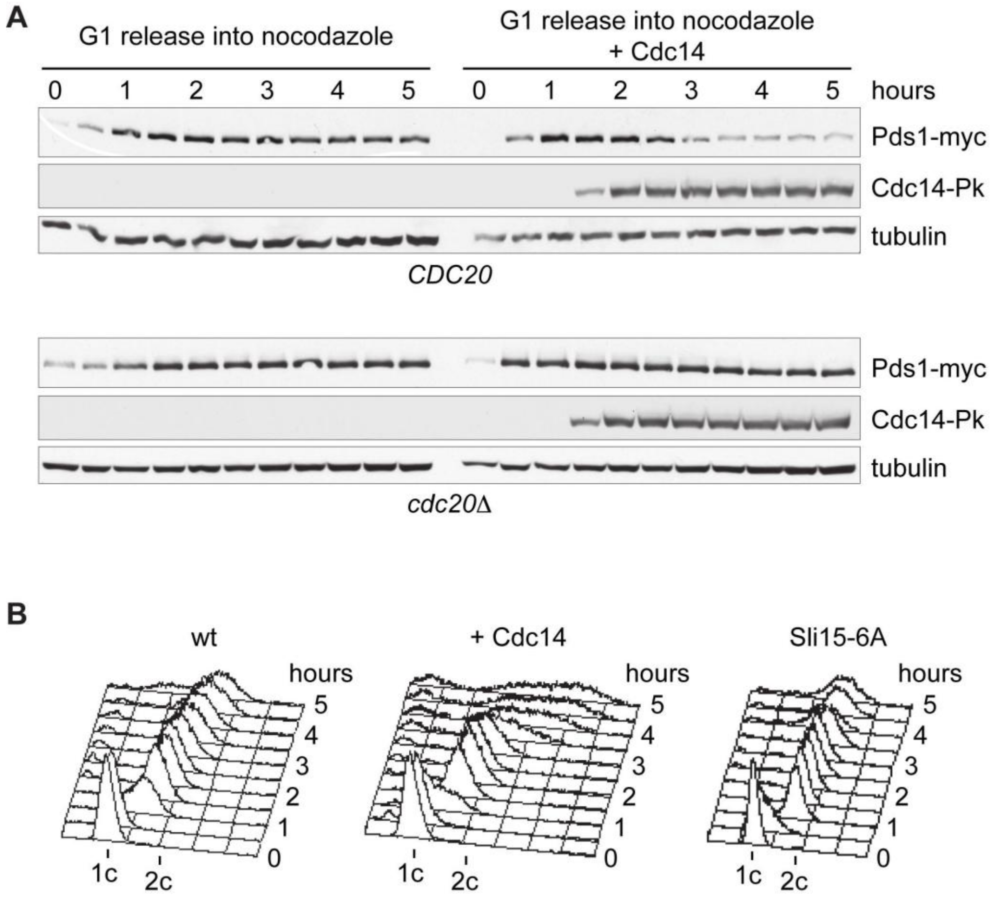

Figure S1. Ectopic Cdc14 Expression, but Not Sli15-6A, Overcomes a Nocodazole-Imposed Mitotic Checkpoint Arrest

(A) Ectopic Cdc14 expression overcomes a nocodazole-imposed mitotic arrest. Cells were synchronized in G1 by α-factor treatment and released into nocodazole-containing medium. Cells harboring the *MET3-CDC20* allele were grown in synthetic medium lacking methionine and were shifted to YP medium supplemented with 2 mM methionine to repress Cdc20 expression at the time of release. One hour after release, Cdc14 expression was induced under control of the *GAL1* promoter in half of the cultures. After 1½ hours, α-factor was re-added to prevent possible securin re-accumulation in the next cell cycle. Securin remained stable throughout the arrest in cells that did not express Cdc14, but was degraded in response to Cdc14 induction. Cdc14-imposed securin destruction depended on the APC activator Cdc20, and did not occur in cells depleted of Cdc20. This is consistent with the possibility that ectopic Cdc14 downregulates the mitotic checkpoint response to nocodazole treatment.

(B) Sli15-6A does not overcome a nocodazole-imposed mitotic checkpoint arrest. As (A), but α-factor was not re-added. Re-budding and re-replication in the presence of nocodazole was observed in response to ectopic Cdc14 expression, but not in *sli15-6A* cells.

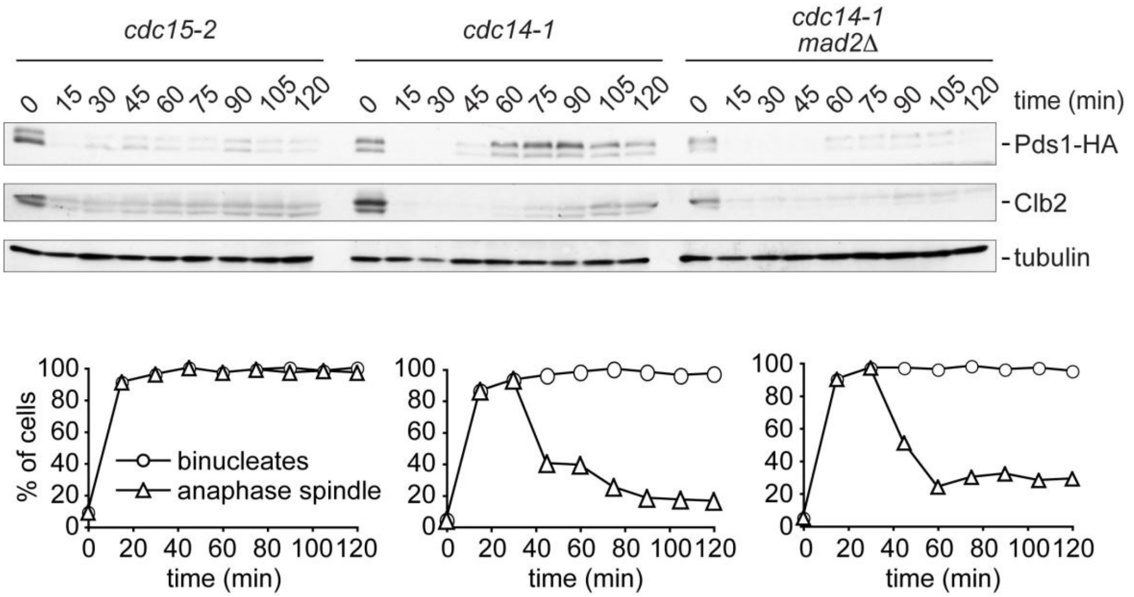

Figure S2. Checkpoint-Dependent Securin and Clb2 Reaccumulation in *cdc14-1* Mutant Anaphase

Cells of the indicated genotypes were arrested in metaphase by depletion of Cdc20 under control of the *GAL1* promoter. The temperature was raised to 37ºC to inactivate the *cdc14-1* and *cdc15-2* alleles, respectively, and cells were released into synchronous anaphase progression by Cdc20 re-induction. Samples at the indicated times after release were processed for Western blotting against securin (Pds1) and Clb2. Tubulin served as a loading control. Anaphase progression was monitored by indirect immunofluorescence staining of tubulin, and by scoring nuclear division. Anaphase spindles break down soon after elongation in *cdc14-1* cells due to defective spindle midzone assembly [5, 16-18].

cdc15-2 cells served as a control in which Cdc14 is activated during early anaphase. In these cells, securin was largely degraded and Clb2 levels decreased to a lower steady state level in response to Cdc20 re-induction. Note that Cdc20 is thought to be insufficient for complete Clb2 destruction, which requires activation of APC^{Cdh1}. Cdh1 remains inactive in *cdc15-2* mutants cells due to disruption of the mitotic exit network.

In *cdc14-1* mutant cells, we observed efficient securin and Clb2 destruction in response to Cdc20 re-induction, initially to levels lower than those observed in *cdc15-2* cells. This might be because Cdc14 is responsible for limiting the activity of Cdc20 during anaphase progression. After 45 min, securin, and later also Clb2 started to re-accumulate. Securin and Clb2 re-accumulation depended on a functional mitotic checkpoint and was reduced in the absence of Mad2. The relatively long time (45 min) until checkpoint-dependent securin re-accumulation in *cdc14-1* mutant anaphase cells might be due to the high levels of Cdc20 after its *GAL1* promoter-driven re-induction that must be overcome by the mitotic checkpoint before securin can re-accumulate.

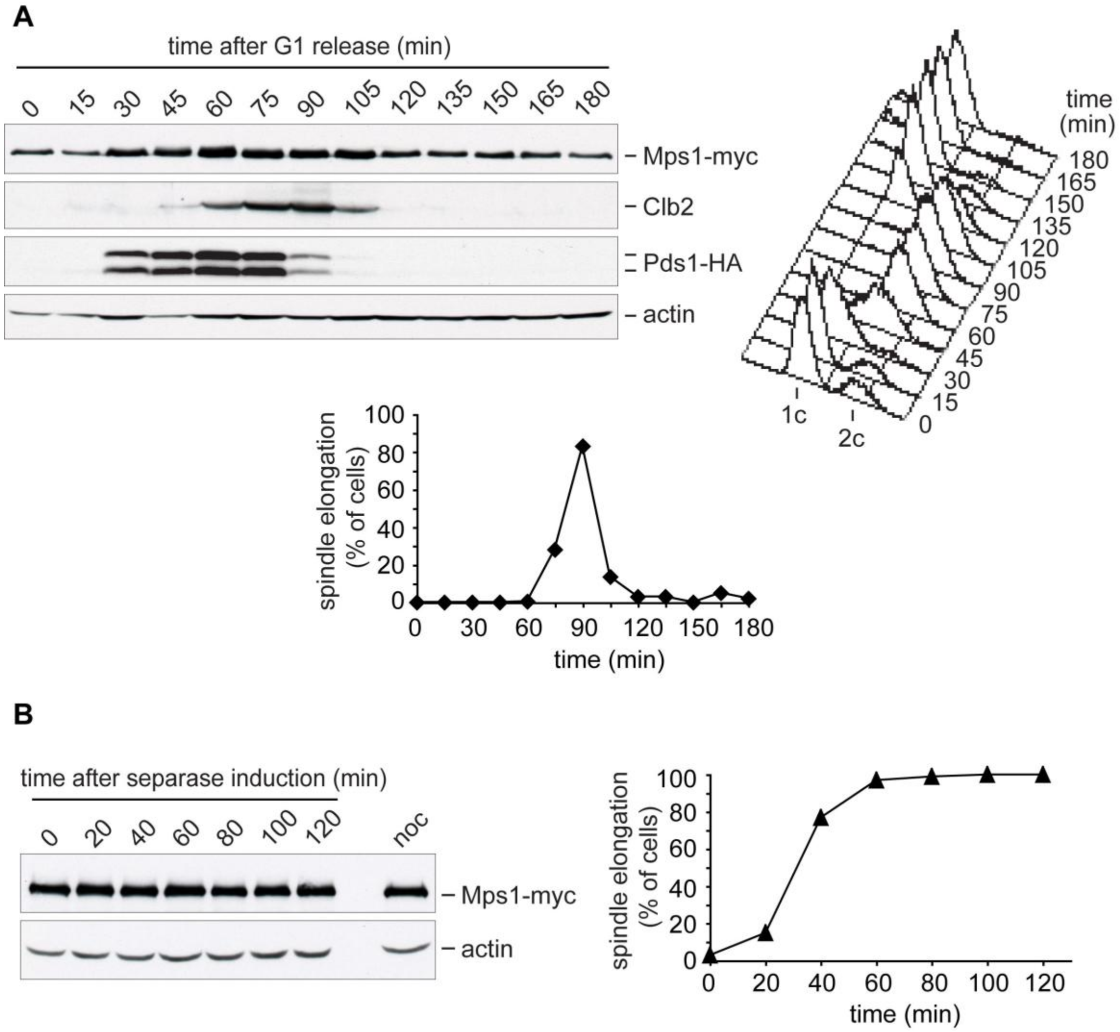

Figure S3. Mitotic Checkpoint Inactivation at Anaphase Onset in the Presence of High Mps1 Levels

(A) Mps1 levels decline late during mitotic exit, after anaphase is complete. Cells were arrested in G1 by α-factor treatement and released into synchronous cell cycle progression. At 60 min, α-factor was added back to halt cell cycle progression in the next G1 phase. Cell extracts were prepared at the indicated time points and protein levels of Mps1, fused to a myc epitope tag for detection, Clb2 and securin (Pds1), fused to an HA epitope tag, were analyzed by Western blotting. Actin served as a loading control. Cell cycle progression was monitored by FACS analysis of DNA content and indirect immunofluorescence staining of tubulin. At the time of anaphase onset, evident by securin destruction and spindle elongation, little change to Mps1 levels was observed. Only at later timepoints, Mps1 levels decreased.

(B) Unaltered Mps1 levels while the mitotic checkpoint is inactivated in response to separase expression in mitotically arrested cells. Separase expression was induced in cells arrested in metaphase by Cdc20 depletion, as in Figure 1. Spindle elongation was monitored by tubulin staining. Mps1 levels, as detected by Western blotting, did not change during the course of the experiment.

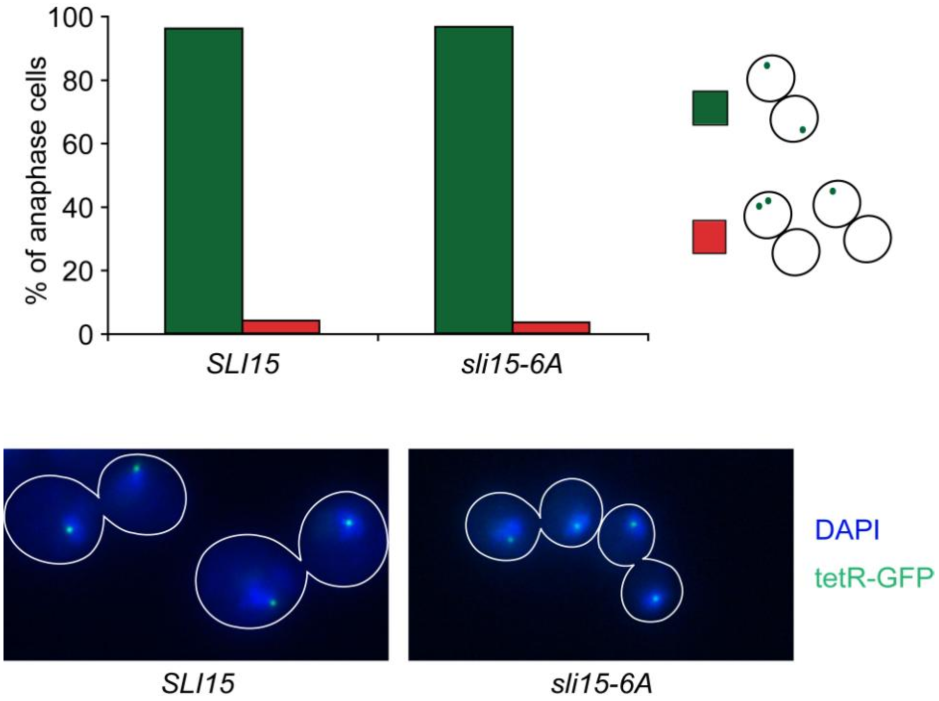

Figure S4. Sli15-6A Is Proficient in Supporting Chromosome Biorientation
Wild type and *sli15-6A* cells were arrested in mitosis using the spindle poison nocodazole that disrupts kinetochore microtubule interactions. After arrest for 2 hours, cells were released into fresh medium without nocodazole to resume mitotic spindle formation, chromosome biorientation and sister chromatid segregation. One hour after release, 52.5% and 61% of cells in the two cultures had entered anaphase, respectively, seen as binucleated cells by staining with 4',6-diamidino-2-phenylindole (DAPI). Correct segregation of sister chromatids of chromosome 5, marked at the *URA3* locus using the tetOs/tetR-GFP system, to opposite cell poles in both cultures demonstrates the efficiency of Aurora B kinase-dependent chromosome biorientation in wild type and *sli15-6A* cells.

Table S1. Yeast Strains Used in This Study (All Strains Were of w303 Background)

Figure 1

Y360	*MAT*a *MET-HA$_3$-CDC20::TRP1 GAL-flag-ESP1-CBD::TRP1(x6) MAD1-HA$_3$::URA BUB1-3eGFP::HIS3*
Y657	*MAT*a *MET-HA$_3$-CDC20::TRP1 scc1Δ::HIS3 SCC1-TEV268-HA$_3$::LEU2 GAL-NLS-myc$_9$-TEVprotease-NLS$_2$::TRP1(x10) GAL-CDC14-Pk$_3$::HIS3 MAD1-HA$_3$::URA3*
Y720	*MAT*a *MET-HA$_3$-CDC20::TRP1 SCC1-TEV268-Pk$_3$::LEU2 GAL-NLS-myc$_9$-TEVprotease-NLS$_2$::TRP1(x10) GAL-CDC14-Pk$_3$::URA3 BUB1-3eGFP::HIS3*
Y721	*MAT*a *MET-HA$_3$-CDC20::TRP1 SCC1-TEV268-Pk$_3$::LEU2 GAL-NLS-myc$_9$-TEVprotease-NLS$_2$::TRP1(x10) BUB1-3eGFP::HIS3*
Y850	*MATα MET-HA$_3$-CDC20::TRP1 scc1Δ::HIS3 SCC1-TEV268-HA$_3$::LEU2 GAL-NLS-myc$_9$-TEVprotease-NLS$_2$::TRP1(x10) MAD1-HA$_3$::URA3*
Y851	*MAT*a *MET-HA$_3$-CDC20::TRP1 GAL-flag-ESP1-CBD::TRP1(x6) MAD1-HA$_3$::URA3*

Figure 2

Y3025	*MAT*a *cdc14-1 MAD1-HA$_3$::URA3 PDS1-myc$_{18}$::TRP1*
Y3026	*MAT*a *cdc15-2 MAD1-HA$_3$::URA3 PDS1-myc$_{18}$::TRP1*
Y3147	*MAT*a *cdc14-1 MAD1-HA$_3$::URA3 PDS1-myc$_{18}$::TRP1 mad2Δ::LEU2*
Y4057	*MAT*a *cdc14-1 MAD1-HA$_3$::URA3 PDS1-2A-myc$_{18}$::TRP1*

Figure 3

Y2669	*MAT*a *scc1-73 PDS1-HA$_6$::URA3, sli15-6A-HA$_6$::HIS3*
Y3774	*MAT*a *scc1-73 PDS1-HA$_6$::HIS3 GAL-CDC14-Pk$_3$::URA3*
Y3789	*MAT*a *PDS1-HA$_6$::HIS3*

Figure 4

Y850	As above
Y3595	*MATα MET-HA$_3$-CDC20::TRP1 scc1Δ::HIS3 SCC1-TEV268-HA$_3$::LEU2 GAL-NLS-myc$_9$-TEVprotease-NLS$_2$::TRP1(x10) MAD1-HA$_3$::URA3 sli15-6A-HA$_6$::HIS3*

Figure S1

Y2400	*MAT*a *PDS1-myc$_{18}$::TRP1 CDC14-HA$_6$::HIS3*
Y4065	*MAT*a *PDS1-myc$_{18}$::TRP1 GAL1-CDC14-Pk$_3$::LEU2*
Y1851	*MAT*a *MET3-HA-CDC20::TRP1 PDS1-myc$_{18}$::URA3 CDC14-HA$_6$::HIS3*
Y2761	*MAT*a *MET3-HA-CDC20::TRP1 PDS1-myc$_{18}$::URA3 CDC14-HA$_6$::HIS3 GAL1-CDC14-Pk$_3$::LEU2*
K699	*MAT*a (w303 'wild type' background)
Y3594	*MAT*a *sli15-6A-HA$_6$::HIS3*

Figure S2

**Y3967**	***MAT*α *GAL-CDC20::URA3 cdc14-1 PDS1-HA$_6$::HIS3***
**Y3968**	***MAT*α *GAL-CDC20::URA3 cdc15-2 PDS1-HA$_6$::HIS3***
**Y3969**	***MAT*a *GAL-CDC20::URA3 cdc14-1 mad2Δ::LEU2 PDS1-HA$_6$::HIS3***

Figure S3

**Y2671**	***MAT*a *PDS1-HA$_6$::HIS3 MPS1-myc$_{18}$::TRP1***
**Y3760**	***MAT*a *MET-HA$_3$-CDC20::TRP1 GAL-flag-ESP1-CBD::TRP1(x6) MAD1-HA$_3$::URA3 MPS1-myc$_{18}$::LEU2***

Figure S4

**K6745**	***MAT*a *TetOs::URA3 TetR::LEU2***
**Y3947**	***MAT*a *sli15-6A-HA$_6$::HIS3 TetOs::URA3 TetR::LEU2***
